# Workplace Preferences and Expectations of Nursing Students: Gender and Generational Differences

**DOI:** 10.1155/nrp/3221299

**Published:** 2026-02-12

**Authors:** Mireia Subirana-Casacuberta, Guadalupe Sanchez-Rueda, Núria Codern-Bové, Maria Aurelia Sanchez-Ortega, Montserrat Martín-Baranera, Lena Ferrus-Estopà

**Affiliations:** ^1^ Department of Nursing, Autonomous University of Barcelona (UAB), Bellaterra, Spain, uab.cat; ^2^ Research Group 2021-SGR-01542, Parc Taulí University Hospital, Parc Taulí Research and Innovation Institute (I3PT-CERCA), Sabadell, Spain; ^3^ Research Group on Complex Health Diagnoses and Interventions From Occupation and Care (OCCARE), University School of Nursing and Occupational Therapy of Terrassa (EUIT), Autonomous University of Barcelona (UAB), Terrassa, Spain, uab.cat; ^4^ Department of Professional Foundations and Health Management, Faculty of Health Sciences and Well-Being, University of Vic–Central University of Catalonia (UVic-UCC), Vic, Spain; ^5^ Department of Preventive Medicine and Public Health, Autonomous University of Barcelona (UAB), Bellaterra, Spain, uab.cat; ^6^ Chair of Management, Direction, and Health Administration, Autonomous University of Barcelona (UAB), Bellaterra, Spain, uab.cat

**Keywords:** environment design, gender, motivation, nursing students, workforce issues

## Abstract

**Background:**

The global nursing shortage has become a critical issue, worsened by poor working conditions, demographic shifts, and the COVID‐19 pandemic. Understanding nursing students’ preferences and expectations is crucial for improving workforce retention, guiding decision‐making, and enhancing job satisfaction. This study aimed to explore nursing students’ preferences and expectations regarding working environment dimensions and analyze differences by gender and generation.

**Methods:**

A quantitative descriptive cross‐sectional study was conducted using an online survey targeting 1277 nursing students from Catalan universities between March 27 and June 26, 2023. Convenience sampling was applied. Preferences and expectations for all items were recorded on a scale from 0 (minimum) to 10 (maximum), and responses were regrouped into three categories: low (0–4), moderate (5–7), and high (8–10). Data analysis was conducted using R (version 4.3.0) under RStudio (version 2023.03.0). The Kolmogorov–Smirnov test confirmed non‐normal distribution of continuous variables. Comparisons by gender and generation were made using chi‐square tests, with significance set at *p* < 0.05. Cronbach’s alpha was used to assess internal consistency for the questionnaire items, confirming the reliability of the survey dimensions.

**Results:**

A significant gap was found in high category (scores 8–10) between nursing students’ preferences, which exceeded 80% for all working environment dimension items and their expectations, which remained below 50% in all cases, indicating a noticeable disparity.

**Conclusions:**

The study highlights a significant discrepancy between nursing students’ high preferences (> 80%) for working environment dimensions and their low expectations (< 50%) of achieving them. This gap suggests that improving work conditions and recognition could stabilize the nursing workforce.

## 1. Introduction

Global nursing shortages strain healthcare systems worldwide, jeopardizing care quality and patient safety [1–3]. Aging populations and demographic shifts increase demand for nurses, exacerbated by inefficient policies and organizational structures [3, 4]. In Spain, the 2008 economic crisis and, more recently, the COVID‐19 pandemic have worsened working conditions, contributing to increased burnout and job dissatisfaction among nurses [1, 5].

Many nursing students gradually lose motivation as they progress through their studies, contributing to persistently high dropout rates [6]. Evidence highlights that targeted academic mentoring, financial support, and burnout prevention programs increase student satisfaction and retention [6, 7]. For new graduates, stable employment and structured residency or mentoring schemes ease the transition into practice and help reduce early turnover [8]. Comprehensive strategies, combining academic and workplace support, play a critical role in sustaining interest in nursing and improving long‐term retention.

Nurses play a pivotal role in healthcare, providing essential care, promoting health, and preventing illness. The World Health Organization (WHO) emphasizes that a robust nursing workforce is vital for achieving universal health coverage and meeting diverse community needs [9]. The “Health and Care Workforce in Europe: Time to Act” report presents 10 concrete strategies to address workforce challenges, but stresses that success depends not only on policy formulation but also on tailoring each measure to local needs, resources, and organizational structures across Europe’s diverse contexts [10]. Despite policy efforts, continued burnout and shortages threaten care quality and system sustainability. Solutions must extend beyond recruitment, emphasizing nurse leadership in workforce planning and policy design, as nurses offer essential frontline insights [11].

Spain, particularly Catalonia, illustrates evolving policy responses to nursing shortages, with initial concerns about high student dropout rates and low retention of new graduates [12]. Recent initiatives have expanded the single bachelor’s‐level nursing education program in Spain and have increased student enrollment, primarily in Catalonia. However, without addressing underlying factors contributing to attrition and early career abandonment, these measures risk limited impact. Understanding nursing students’ workplace preferences is thus crucial for developing targeted strategies to enhance satisfaction and retention [6].

Herzberg’s motivation‐hygiene theory provides the conceptual framework for this study, as it is meaningful for analyzing workplace preferences and expectations of nursing students. Motivational factors, such as opportunities for professional growth, recognition, and responsibility, foster satisfaction. Hygiene factors, like working conditions, schedule flexibility, and adequate resources, prevent dissatisfaction [13]. Generational differences also influence expectations, with Generation Z (Gen Z) students prioritizing work–life balance, flexible schedules, and personal development, elements central to their retention [7].

This study examines the preferences and expectations of nursing students concerning workplace choices in Catalonia, using Herzberg’s motivation‐hygiene theory as the conceptual framework, and analyzes distinctions by gender and generation.

## 2. Materials and Methods

### 2.1. Study Design and Sample

We conducted an exploratory cross‐sectional study, adhering to the STROBE guidelines for reporting observational research [14]. The survey design followed the adapted version of Guide No. 87 by the Association for Medical Education in Europe [15]. The project was approved by the Ethics Committee of the *Universitat Autònoma de Barcelona* (CEEAH‐6325) in March 2023.

The target population consisted of all 9387 students enrolled in nursing program across 18 institutions in Catalonia during the 2022–2023 academic year. These institutions included six public universities (with affiliated centers) and three private universities. Enrollment was distributed as follows: 45% in public universities, 46% in affiliated centers, and 9% in private institutions.

Recruitment took place between March 27 and June 26, 2023. Participation was voluntary and anonymous. Students were eligible if they completed all items in the online questionnaire. A minimum sample of 370 respondents was required to estimate a 50% proportion with 95% confidence and ±5% precision.

### 2.2. Survey Development

The survey was developed using a rigorous, structured process that integrated a comprehensive literature review with stakeholder input [15]. First, relevant concepts, theoretical frameworks, and candidate items were identified through an extensive review of the literature. Subsequently, individual semistructured interviews lasting 45–60 min were conducted with key stakeholders to explore how nursing students and professionals conceptualize the work environment. Interview participants included five students (from public, affiliated, and private universities), seven nursing professors, two human resources managers in healthcare, and one hospital manager. We selected participants purposively for their experience in education and nursing practice. Interviews were recorded, transcribed verbatim, and supplemented with field notes.

We developed the initial version of the survey (QZero) from the findings and literature insights. Validation proceeded in three phases:

1. Feasibility: assessed through expert reviews and pilot testing with students and professors.
2. Validity: face validity ensured item clarity and relevance through expert evaluation; content validity was evaluated using the content validity index (CVI), with acceptable item and scale‐level scores (I‐CVI ≥ 0.78, S‐CVI > 0.8); construct validity was supported by the alignment of survey items with theoretical dimensions. Criterion validity was not assessed due to the absence of a gold standard.
3. Reliability: we tested internal consistency using Cronbach’s alpha for each work environment dimension. Test‐retest reliability and inter‐rater reliability were not applicable.

This structured process validated the survey’s rigor and reliability [15, 18] leading to the final version (SFV).

### 2.3. Survey Structure

The SFV included four sections: Section 1: introduction: outlined study objectives, informed consent, and confidentiality assurances. Section 2: participant information: captured course and institution details. Section 3: workplace preferences and expectations. Participants scored preferences and expectations for six workplace dimensions on a 0–10 scale: nursing practice (6 items); professional development (5); well‐being and work climate (7); work schedules (3); resources (3), and employment conditions and pay (4). Table 1 provides working environment dimension items and the corresponding Cronbach’s alpha values for each dimension. Section 4: sociodemographic data: included gender, age, educational background, parents’ education, and willingness to choose nursing again.

**Table 1:** Working environment dimensions and Cronbach’s alpha for preferences and expectations.

	**Working environment dimensions**	**Cronbach’s alpha preference**	**Cronbach’s alpha expectation**

Motivational factors	1. Nursing practice (6 items)	0.787	0.860
	2. Professional development opportunities (5 items)	0.802	0.881
	3. Well‐being and work environment (7 items)	0.842	0.916

Hygiene factors	4. Schedules (3 items)	0.769	0.883
	5. Resources (3 items)	0.825	0.826
	6. Terms of employment and remuneration (4 items)	0.868	0.750

The pilot version was tested for clarity. A broad sample of Catalan nursing students completed the 12–15 min survey across platforms. A QR code linked participants to a secure SurveyMonkey form, shared through institutional emails and social media. Weekly follow‐ups encouraged participation. All 18 university directors collaborated by providing enrollment figures. A professor guided validation of the QZero instrument, while project‐promoting professors supported outreach.

### 2.4. Variables and Data Sources

Key variables included gender, generation, and workplace‐related preferences and expectations. Following Herzberg’s motivation‐hygiene theory [13]: motivational factors: nursing practice, professional development, well‐being, and hygiene factors: schedules, resources, and terms of employment. Preferences reflected valued job attributes; expectations referred to anticipated outcomes based on past experiences or perceptions.

Gender was self‐reported (men, women, and others). Generation was based on the birth year: Gen Z (born 1997 or later) and pre‐Gen Z (born before 1997). To reduce bias, we included all eligible institutions, ensured sample diversity, and applied rigorous statistical analysis.

### 2.5. Questionnaire Reliability

Cronbach’s alpha was calculated for the total sample and subgroups to evaluate internal consistency, confirming the reliability of each survey dimension. Values for the six workplace dimensions, both for preferences and expectations (Table 1), indicated satisfactory internal consistency (all > 0.70), validating the instrument’s reliability in this context [16].

### 2.6. Data Analysis

We conducted the analysis in R (version 4.3.0) using RStudio (version 2023.03.0 Build 386). We checked the distribution of continuous variables with the Kolmogorov–Smirnov test, confirming non‐normality. Continuous variables are presented as medians and interquartile ranges (IQR) reported as the 25th and 75th percentiles. Categorical variables are summarized as absolute frequencies and percentages.

Preference and expectation scores (0–10 scale) were grouped into three categories: low (0–4), moderate (5–7), and high (8–10). For gender and generation comparisons, we used recoded variables and chi‐square tests, with statistical significance set at *p* < 0.05.

### 2.7. Ethical Consideration

The UAB Ethics Committee approved the study (CEEAH‐6325), along with participating academic institutions. Participation was voluntary, and all data were collected anonymously and confidentially. Participants gave informed consent for their data to be used only for research purposes.

## 3. Results

This study analyzed the preferences and expectations of nursing students regarding six key workplace dimensions, classified according to Herzberg’s motivation‐hygiene theory [13], as motivational factors (nursing practice, professional development opportunities, well‐being and, work environment) and hygiene factors (work schedules, resources, employment terms, and remuneration).

### 3.1. Participant Characteristics

A total of 1277 students completed the survey, yielding a 13.6% response rate. Participant characteristics (*n* = 1277) are detailed in Table 2. Most were Gen Z women (78%, 83%) combining studies with employment (55%), primarily from public university‐affiliated centers (61%). Binary gender analyses used *n* = 1262.

**Table 2:** Participant characteristics^∗^.

Age (years)	
Mean (SD); range	23; 18–56
Generation	
Generation Z	996 (78.0)
Pre‐Gen Z	281 (22.0)
Gender identity	
Women	1046 (83.0)
Men	216 (17.0)
Nonbinary	9 (0.7)
Not specified	6 (0.5)
Binary gender analyses (*n* = 1262)	
Women	1046 (82.9)
Men	216 (17.1)
Employment status	
Employed (total)	702 (55.0)
Healthcare sector	210 (30.0)
Nonhealthcare	492 (25.0)
Not employed	575 (44.0)
Institution type	
Public university‐affiliated centers	779 (61.0)
Public universities	434 (34.0)
Private universities	64 (5.0)

^∗^Continuous variable is expressed as the median with the interquartile range (IQR), reported as the 25th and 75th percentiles. Categorical variables are summarized as absolute frequencies and percentages.

### 3.2. Overall Preferences and Expectations

Preferences and expectations were analyzed using a clear, standardized reporting format [17] and disaggregated by total sample, gender, and generation, as recommended. Tables 3, 4, and 5 present the distribution of responses; Table 3 presents an overview of preferences and expectations for the entire sample, while Tables 4 and 5 display analyses stratified by gender and generation, respectively.

**Table 3:** Preference and expectation of working environment dimensions.

		**Preference**			**Expectation**	
	**Total *n* = 1277**			**Total *n* = 1277**		
	**Low (%)**	**Moderate (%)**	**High (%)**	**Low (%)**	**Moderate (%)**	**High (%)**
*Motivational factors*						
1. Nursing practice (6 items):						
In the institution where I work can take care of the people under my charge according to professional values	0.0	7.8	92.2	7.0	51.2	41.8
Autonomous nursing practice is being recognized within the organization	0.1	3.7	96.2	12.8	55.2	32.0
Institution recognizes economically and professionally the nurses for the competencies they develop and their level of training	0.2	3.1	96.7	26.2	54.7	19.1
Institution promotes reflective practice	0.7	17.1	82.2	14.7	57.0	28.3
Institution promotes evidence‐based practice	0.3	11.2	88.5	5.7	40.5	53.8
Institution promotes teamwork to contribute to patient safety	0.1	5.7	94.2	7.8	41.8	50.4
2. Professional development opportunities (5 items):						
Institution recognizes professional development	0.2	5.2	94.6	12.5	56.4	31.1
Institution provides opportunities for training and learning to advance professionally	0.5	4.1	95.5	8.3	46.8	44.9
Institution promotes research, innovation, and professional development through scholarships, grants, and awards for studies/training in other institutions	0.6	12.5	86.8	19.6	55.3	25.1
Institution recognizes economically and professionally the specializations	0.5	4.5	95.1	28.2	51.2	20.6
Institution recognizes economically and professionally the advanced nursing practice	0.5	4.2	95.3	26.8	49.9	23.3
3. Well‐being and work environment (7 items):						
Institution provides individualized welcoming	1.4	13.9	84.7	22.7	51.3	26.0
Institution supports novice professionals	0.2	5.2	94.6	27.4	50.8	21.8
Institution tracks my progress in an environment of trust and confidentiality	0.9	12.8	86.3	23.9	52.5	23.6
Institution provides an environment of trust and confidentiality to ask and share doubts	0.3	4.3	95.4	19.3	49.5	31.2
In situations of conflict, nursing leaders (supervisors, coordinators, etc.) listen to me and consider my point of view	0.2	3.4	96.3	20.8	54.0	25.1
Institution offers psychological support, if needed, for stress and anxiety management	0.5	6.8	92.6	39.7	42.7	17.6
Institution provides preventive measures, if needed, for stress and anxiety management	1.2	8.7	90.1	40.0	44.9	15.1
*Hygienic factors*						
4. Schedules (3 items):						
Institution offers me contracts with a weekly working schedule that allows for a work–life balance	0.2	3.3	96.5	43.5	43.1	13.3
Institution allows me to choose schedules and shifts that are adapted to my needs	0.4	5.1	94.5	53.2	36.5	10.3
Institution provides me with the work schedule in advance so that I can organize my personal life	0.5	3.0	96.6	37.3	43.9	18.9
5. Resources (3 items):						
Number of nurses in the institution allows patients safety and quality care	0.2	2.5	97.3	33.8	48.0	18.2
Institution has the necessary technological resources for safety and quality care	0.3	4.4	95.3	14.2	51.1	34.7
Institution has the necessary material resources (instruments, care materials, beds, etc.) available to safely and effectively care for the individuals attended to	0.3	3.0	96.7	11.5	50.5	38.0
6. Terms of employment and remuneration (4 items):						
Institution offers me a permanent or long‐term employment contract	0.8	6.0	93.2	41.4	39.9	18.6
Institution offers me the best remuneration based on my competencies and level of responsibility	0.2	3.2	96.5	40.9	44.8	14.3
Institution provides me with a contract that allows me to balance work with external education (master’s degree, preparing for a specialty exam, Ph.D., etc.)	0.4	4.2	95.4	37.4	45.9	16.7
Institution provides me with the opportunity to work extra hours or receive paid overtime	0.8	7.2	92.0	16.5	38.9	44.6

*Note:* Participants’ original ratings, on a scale from 10 (maximum) to 0 (minimum), were categorized into three levels: 0–4 (low preference or expectation); 5–7 (moderate preference or expectation; and 8–10 (high preference or expectation).

**Table 4:** Preference and expectation of working environment dimensions by gender.

	**Participants’ categorized answers^∗^**	**Preference**			**Expectation**		
		**Gender**		**Chi-square test** ***p* value**	**Gender**		**Chi-square test** ***p* value**
		**Women (%)** ***n* = 1046**	**Men (%)** ***n* = 216**		**Women (%)** ***n* = 1046**	**Men (%)** ***n* = 216**	
*Motivational factors*							
1. Nursing practice (6 items):							
In the institution where I work can take care of the people under my charge according to professional values	Low	0.0	0.0	**0.024**	6.5%	8.4%	0.560
	Moderate	7.1	11.6		51.1	51.6	
	High	92.9	88.4		42.4	40.0	
Autonomous nursing practice is being recognized within the organization	Low	0.0	0.5	**< 0.0005**	12.5	13.0	0.978
	Moderate	2.8	7.9		55.4	55.6	
	High	97.2	91.7		32.0	31.5	
Institution recognizes economically and professionally the nurses for the competencies they develop and their level of training	Low	0.1	0.9	**< 0.0005**	25.0	31.9	0.077
	Moderate	2.4	6.5		56.1	48.6	
	High	97.5	92.6		18.8	19.4	
Institution promotes reflective practice	Low	0.6	1.4	0.431	13.6	19.4	0.061
	Moderate	17.0	17.1		57.2	56.0	
	High	82.4	81.5		29.2	24.5	
Institution promotes evidence‐based practice	Low	0.3	0.5	0.622	5.6	6.0	0.370
	Moderate	10.9	13.0		39.6	44.4	
	High	88.8	86.6		54.8	49.5	
Institution promotes teamwork to contribute to patient safety	Low	0.1	0.0	0.889	7.7	7.9	0.756
	Moderate	5.8	5.6		42.1	39.4	
	High	94.1	94.4		50.2	52.8	
2. Professional development opportunities (5 items):							
Institution recognizes professional development	Low	0.1	0.5	0.305	12.0	15.7	0.289
	Moderate	5.0	6.5		57.0	53.2	
	High	94.9	93.1		31.1	31.0	
Institution provides opportunities for training and learning to advance professionally	Low	0.3	1.4	0.067	8.1	9.3	0.758
	Moderate	3.8	5.1		46.5	47.7	
	High	95.9	93.5		45.4	43.1	
Institution promotes research, innovation, and professional development through scholarships, grants, and awards for studies/training in other institutions	Low	0.6	0.9	**0.021**	19.0	21.8	0.644
	Moderate	11.4	18.1		55.5	54.2	
	High	88.0	81.0		25.4	24.1	
Institution recognizes economically and professionally the specializations	Low	0.3	0.9	0.389	27.2	32.4	0.275
	Moderate	4.5	4.2		52.0	47.2	
	High	95.2	94.9		20.8	20.4	
Institution recognizes economically and professionally the advanced nursing practice	Low	0.5	0.5	0.942	25.6	31.9	**0.027**
	Moderate	4.1	4.6		51.6	41.7	
	High	95.4	94.9		22.8	26.4	
3. Well‐being and work environment (7 items):							
Institution provides individualized welcoming	Low	1.2	2.3	**0.024**	23.3	19.0	0.377
	Moderate	12.8	19.0		50.8	53.2	
	High	85.9	78.7		25.9	27.8	
Institution supports novice professionals	Low	0.3	0.0	0.191	27.0	27.8	0.722
	Moderate	4.7	7.4		51.4	48.6	
	High	95.0	92.6		21.6	23.6	
Institution tracks my progress in an environment of trust and confidentiality	Low	1.0	0.9	0.936	23.5	24.1	0.264
	Moderate	12.5	13.4		53.4	48.1	
	High	86.5	85.6		23.0	27.8	
Institution provides an environment of trust and confidentiality to ask and share doubts	Low	0.3	0.5	0.384	18.5	21.3	0.145
	Moderate	4.0	6.0		50.9	43.5	
	High	95.7	93.5		30.7	35.2	
In situations of conflict, nursing leaders (supervisors, coordinators, etc.) listen to me and consider my point of view	Low	0.1	0.5	0.369	20.0	22.7	0.263
	Moderate	3.3	4.2		55.2	49.1	
	High	96.7	95.4		24.9	28.2	
Institution offers psychological support, if needed, for stress and anxiety management	low	0.7	0.0	**0.021**	40.4	33.8	0.129
	moderate	5.9	10.6		42.5	44.9	
	high	93.4	89.4		17.0	21.3	
Institution provides preventive measures, if needed, for stress and anxiety management	Low	1.1	1.9	**0.004**	40.9	33.8	0.150
	Moderate	7.6	14.4		44.3	49.5	
	High	91.3	83.8		14.8	16.7	
*Hygienic factors*							
4. Schedules (3 items):							
Institution offers me contracts with a weekly working schedule that allows for a work–life balance	Low	0.3	0.0	**0.013**	43.9	41.7	0.280
	Moderate	2.7	6.5		43.5	41.7	
	High	97.0	93.5		12.6	16.7	
Institution allows me to choose schedules and shifts that are adapted to my needs	Low	0.4	0.5	**p** < 0.0005	53.6	51.4	0.604
	Moderate	3.9	11.1		36.5	36.6	
	High	95.7	88.4		9.8	12.0	
Institution provides me with the work schedule in advance so that I can organize my personal life	Low	0.4	0.9	0.150	38.0	32.9	0.079
	Moderate	2.6	4.6		44.3	43.1	
	High	97.0	94.4		17.8	24.1	

5. Resources (3 items):							
Number of nurses in the institution allows patients safety and quality care	Low	0.2	0.0	0.081	33.4	34.7	0.923
	Moderate	2.1	4.6		48.4	47.7	
	High	97.7	95.4		18.3	17.6	
Institution has the necessary technological resources for safety and quality care	Low	0.3	0.5	0.060	14.0	14.4	0.611
	Moderate	3.8	7.4		51.7	48.1	
	High	95.9	92.1		34.3	37.5	
Institution has the necessary material resources (instruments, care materials, beds, etc.) available to safely and effectively care for the individuals attended to	Low	0.2	0.9	**0.030**	11.5	9.7	0.289
	Moderate	2.6	5.1		49.7	55.6	
	High	97.2	94.0		38.8	34.7	
6. Terms of employment and remuneration (4 items):							
Institution offers me a permanent or long‐term employment contract	Low	0.6	1.9	**0.038**	42.3	37.5	**0.010**
	Moderate	5.4	8.3		40.6	36.6	
	High	94.0	89.8		17.1	25.9	
Institution offers me the best remuneration based on my competencies and level of responsibility	Low	0.2	0.5	0.696	41.2	39.8	0.309
	Moderate	3.2	3.7		45.2	42.6	
	High	96.6	95.8		13.6	17.6	
Institution provides me with a contract that allows me to balance work with external education (master’s degree, preparing for a specialty exam, Ph.D., etc.)	Low	0.5	0.0	0.209	38.9	30.6	0.057
	Moderate	3.8	6.0		45.0	49.5	
	High	95.7	94.0		16.1	19.9	
Institution provides me with the opportunity to work extra hours or receive paid overtime	Low	0.6	1.9	0.021	16.8	15.3	0.585
	Moderate	6.4	10.2		39.4	37.0	
	High	93.0	88.0		43.9	47.7	

^∗^Participants’ original ratings, on a scale from 10 (maximum) to 0 (minimum), were categorized into three levels: 0–4 (low preference or expectation); 5–7 (moderate preference or expectation; and 8–10 (high preference or expectation). To facilitate interpretation, *p* values ≥ 0.05 are highlighted in bold and the corresponding cell is shaded.

**TABLE 5:** Preference and expectation of working environment dimensions by generation.

	**Participants’ categorized answers^∗^**	**Preference**			**Expectation**		
		**Generation**		**Chi-square test** ***p* value**	**Generation**		**Chi-square test** ***p* value**
		**Gen Z (%)**	**Pre-Gen Z (%)**		**Gen Z (%)**	**Pre-Gen Z (%)**	
		***n* = 995**	***n* = 282**		**n = 995**	**n = 282**	
*Motivational factors*							
1. Nursing practice (6 items):							
In the institution where I work can take care of the people under my charge according to professional values	Low	0.0	0.0	0.434	5.7	11.3	**0.002**
	Moderate	8.2	6.7		50.8	52.5	
	High	91.8	93.3		43.5	36.2	
Autonomous nursing practice is being recognized within the organization	Low	0.1	0.0	0.415	11.8	16.3	**0.048**
	Moderate	4.0	2.5		56.8	49.6	
	High	95.9	97.5		31.5	34.0	
Institution recognizes economically and professionally the nurses for the competencies they develop and their level of training	Low	0.1	0.7	0.112	25.0	30.5	0.079
	Moderate	2.8	3.9		56.3	48.9	
	High	97.1	95.4		18.7	20.6	
Institution promotes reflective practice	Low	0.8	0.4	0.178	12.3	23.1	**< 0.0005**
	Moderate	18.0	13.8		57.3	55.9	
	High	81.2	85.8		30.4	21.0	
Institution promotes evidence‐based practice	Low	0.3	0.4	0.668	5.4	6.8	0.302
	Moderate	11.6	9.7		39.8	43.2	
	High	88.1	89.9		54.8	50.0	
Institution promotes teamwork to contribute to patient safety	Low	0.1	0.0	0.720	6.8	11.1	**0.001**
	Moderate	5.9	5.0		40.2	47.5	
	High	94.0	95.0		52.9	41.4	
2. Professional development opportunities (5 items):							
Institution provides opportunities for training and learning to advance professionally	Low	0.5	0.4	0.465	7.5	11.0	**0.011**
	Moderate	3.7	5.3		45.5	51.4	
	High	95.8	94.3		46.9	37.6	
Institution promotes research, innovation, and professional development through scholarships, grants, and awards for studies/training in other institutions	Low	0.6	0.7	0.429	17.7	26.2	**0.005**
	Moderate	13.2	10.3		56.2	52.1	
	High	86.2	89.0		26.1	21.6	
Institution recognizes economically and professionally the specializations	Low	0.5	0.4	0.828	25.5	37.6	**< 0.0005**
	Moderate	4.6	3.9		52.8	45.7	
	High	94.9	95.7		21.7	16.7	
Institution recognizes economically and professionally the advanced nursing practice	Low	0.5	0.7	0.316	23.9	36.9	**< 0.0005**
	Moderate	3.7	5.7		51.3	45.0	
	High	95.8	93.6		24.8	18.1	
Institution provides individualized welcoming	Low	1.6	0.7	0.508	20.4	30.9	**0.001**
	Moderate	13.8	14.5		53.3	44.3	
	High	84.6	84.8		26.3	24.8	
3. Well‐being and work environment (7 items):							
Institution supports novice professionals	Low	0.3	0.0	0.597	25.9	32.6	0.058
	Moderate	5.0	5.7		51.4	48.9	
	High	94.7	94.3		22.7	18.4	
Institution tracks my progress in an environment of trust and confidentiality	Low	1.1	0.4	**0.039**	21.7	31.6	**0.003**
	Moderate	13.9	8.9		54.2	46.5	
	High	85.0	90.8		24.1	22.0	
Institution provides an environment of trust and confidentiality to ask and share doubts	Low	0.2	0.7	0.237	17.6	25.2	**0.003**
	Moderate	4.6	3.2		49.2	50.4	
	High	95.2	96.1		33.2	24.5	
In situations of conflict, nursing leaders (supervisors, coordinators, etc.) listen to me and consider my point of view	Low	0.1	0.7	0.171	19.1	27.0	**0.016**
	Moderate	3.5	3.2		55.1	50.4	
	High	96.4	96.1		25.8	22.7	
Institution offers psychological support, if needed, for stress and anxiety management	Low	0.6	0.4	0.270	39.0	42.2	0.216
	Moderate	6.2	8.9		42.4	43.6	
	High	93.2	90.8		18.6	14.2	
Institution provides preventive measures, if needed, for stress and anxiety management	Low	1.2	1.1	0.554	39.2	42.9	0.507
	Moderate	8.2	10.3		45.3	43.3	
	High	90.6	88.7		15.5	13.8	
*Hygienic factors*							
4. Schedules (3 items):							
Institution offers me contracts with a weekly working schedule that allows for a work–life balance	Low	0.3	0.0	0.387	41.3	51.4	**0.003**
	Moderate	3.0	4.3		45.6	34.4	
	High	96.7	95.7		13.1	14.2	
Institution allows me to choose schedules and shifts that are adapted to my needs	Low	0.5	0.0	0.124	50.7	62.4	**0.001**
	Moderate	5.6	3.2		39.2	27.0	
	High	93.9	96.8		10.2	10.6	
Institution provides me with the work schedule in advance so that I can organize my personal life	Low	0.5	0.4	0.923	33.8	49.6	**< 0.0005**
	Moderate	2.9	3.2		47.0	32.6	
	High	96.6	96.5		19.2	17.7	
5. Resources (3 items):							
Number of nurses in the institution allows patients safety and quality care	Low	0.2	0.0	0.180	31.9	40.8	**0.013**
	Moderate	2.1	3.9		48.9	44.7	
	High	97.7	96.1		19.2	14.5	
Institution has the necessary material resources (instruments, care materials, beds, etc.) available to safely and effectively care for the individuals attended to	Low	0.2	0.7	0.326	10.6	14.9	0.111
	Moderate	2.8	3.5		50.7	50.0	
	High	97.0	95.7		38.8	35.1	
Institution offers me a permanent or long‐term employment contract	Low	0.9	0.4	0.630	38.4	52.1	**< 0.0005**
	Moderate	5.9	6.4		42.5	30.9	
	High	93.2	93.3		19.1	17.0	
6. Terms of employment and remuneration (4 items):							
Institution offers me the best remuneration based on my competencies and level of responsibility	Low	0.3	0.0	0.611	38.8	48.4	**0.006**
	Moderate	3.1	3.6		47.1	36.7	
	High	96.6	96.4		14.1	14.9	
Institution provides me with a contract that allows me to balance work with external education (master’s degree, preparing for a specialty exam, Ph.D., etc.)	Low	0.3	0.7	0.489	35.3	45.0	**0.005**
	Moderate	4.0	5.0		46.8	42.6	
	High	95.7	94.3		17.9	12.4	
Institution provides me with the opportunity to work extra hours or receive paid overtime	Low	0.7	1.1	0.265	14.4	23.8	**0.001**
	Moderate	6.6	9.3		39.3	37.4	
	High	92.7	89.7		46.3	38.8	

^∗^Participants’ original ratings, on a scale from 10 (maximum) to 0 (minimum), were categorized into three levels: 0–4 (low preference or expectation); 5–7 (moderate preference or expectation; and 8–10 (high preference or expectation).To facilitate interpretation, *p* values ≥ 0.05 are highlighted in bold and the corresponding cell is shaded.

The key insights from Table 3 show that preferences for positive workplace conditions were consistently high across all items and dimensions. For most aspects including professional values, teamwork, career advancement opportunities, individualized support, scheduling, and resource availability, more than 90% of students expressed a high preference. Only a few items showed slightly lower consensus, specifically within motivational factor dimensions: (1) nursing practice (institutional promotion of reflective practice and evidence‐based practice), (2) professional development opportunities (support for research, innovation, and professional development through scholarships or external training grants), and (3) well‐being and work environment (individualized welcoming and progress tracking in a confidential, trust‐based setting). Even for these items, more than 80% of students reported high preference. This pattern underscores a broad consensus among nursing students regarding the key components of a desirable work environment. Expectations displayed considerably more variability and remained notably lower across all dimensions (< 55%). In nursing practice, fewer than half expected regular supportive practices such as professional recognition, teamwork, or institutional emphasis on evidence‐based care. Professional development opportunities showed similar trends, with only a minority anticipating significant support for training, recognition, or advancement through scholarships/grants. Well‐being and work environment expectations were moderate at best, with very few confident in personalized support, psychological care, or individualized onboarding/progress tracking. Schedules elicited the greatest pessimism (high ratings < 20% for flexible, personalized, or family‐friendly shifts), followed by resources (limited confidence in adequate staffing/materials for safe care) and terms of employment and remuneration (subdued expectations for stable contracts, advancement, or fair compensation).

### 3.3. Preferences and Expectations by Gender

The gender‐based analysis (Table 4) of preferences offers valuable insights into how nursing students perceive their ideal workplace. In the nursing practice domain, high preference rates are above 90% for both women and men for most items. However, women report significantly higher preferences than men for caring according to professional values (93% vs. 88%), recognition of autonomous nursing practice (97% vs. 92%), and economic and professional recognition within the organization (98% vs. 93%). For the remaining items in this domain, high preference values remain consistently strong across both genders.

In professional development opportunities, high preference exceeds 93% for nearly all items, with no significant differences between genders except for promotion of research, innovation, and professional development through scholarships, grants, and awards, where women report higher preference than men (88% vs. 81%).

In the well‐being and work environment dimension, women express higher preference ratings for individualized welcoming (86% vs. 79%), access to psychological support (93% vs. 89%), and support from nursing professionals within the institution (91% vs. 84%).

For hygienic factors, within the schedules dimension, women highly value having a contract that ensures a weekly schedule compatible with work‐life balance (97% vs. 94%) and choosing schedules adapted to their needs (96% vs. 88%). Regarding resources, all students show nearly unanimous high preference for essential material resources, with women rating access slightly higher (97% vs. 94%). In employment and remuneration, both genders report high preferences, with women showing higher preference for permanent or long‐term contracts (94% vs. 90%).

Analysis of expectations reveals substantial gaps and contrasts between women and men. For motivational factors, overall expectations are low across genders, with no statistically significant differences. In professional development opportunities, a significant difference appears regarding recognition for advanced nursing practice: men (26%) rate this higher than women (23%). In employment and remuneration, men are slightly more likely than women to expect permanent employment contracts (26% vs. 17%). Expectations regarding schedules and resources show no meaningful gender differences.

### 3.4. Preferences and Expectations by Generation

Analysis of Table 5 reveals notable differences between Gen Z and pre‐Gen Z, in their preferences and expectations regarding dimensions of the nursing working environment. While preferences across most items are relatively similar between generations, several expectation items show statistically significant differences, suggesting a generational shift in workplace demands.

Regarding preferences, there are only two items, both within motivational factor dimensions with a significant difference. A higher proportion of pre‐Gen Z nurses (91%) expresses a strong preference for institutions that track their progress in an environment of trust and confidentiality, compared to Gen Z (85%) as well as the recognition of professional development.

In contrast, expectations for the working environment differ notably between generations, especially in several critical areas. A significantly greater proportion of Gen Z respondents expect that institutions will deliver care guided by professional nursing values (44% for Gen Z versus 36% for pre‐Gen Z), recognize autonomous nursing practice within the organization (32% vs. 34%), offer robust opportunities for professional growth and development (47% vs. 38%), promote reflective practice (30% vs. 21.0%), and support teamwork to enhance patient safety (53% vs. 41%). Gen Z nurses also demonstrated significantly higher expectations for all professional development opportunity items and revealed consistent generational differences, with Gen Z reporting higher expectations for individualized welcoming, transparent monitoring of progress, environments of trust, and supportive leadership in conflict situations. Scheduling flexibility also emerged as a key area of significant divergence, as Gen Z nurses were more likely to expect advance notice of schedules, adaptable shifts, and improved work–life balance. Similarly, expectations related to employment stability, remuneration, and opportunities for paid overtime were significantly higher among Gen Z nurses.

### 3.5. Recommendations for Future Research

Future studies should consider integrating Herzberg’s two‐factor theory with the job demands‐resources model to more comprehensively assess how both intrinsic and extrinsic elements interact to influence job satisfaction and occupational well‐being in nursing. Intervention studies should evaluate strategies that increase intrinsic motivators, such as professional development and recognition, and address hygiene factors such as fair remuneration and safe working conditions. Additionally, exploring variations according to level of training, clinical practices, or type of contract could provide valuable insights into how nursing students perceive their work environment. It is recommended to conduct longitudinal studies to examine whether improvements in the work environment influence retention, engagement, and professional development over time.

### 3.6. Strengths and Limitations

This study benefits from a large sample size of 1277 nursing students from Catalan universities. Furthermore, the study successfully identifies a significant gap between students’ high preferences and low expectations regarding workplace conditions and environment, providing critical insights for improving nursing workforce stability.

However, the cross‐sectional design of the study limits the ability to establish causal relationships between preferences and expectations. Additionally, the research relied on self‐reported data from nursing students, as no validated instrument specific to the study’s scope was available. The regional focus on Catalan universities may also restrict the applicability of the findings to other educational or healthcare contexts. Moreover, the use of convenience sampling constitutes a methodological limitation, as it may introduce selection bias and compromise the generalizability of the results. Participants who chose to take part in the study may not be fully representative of the broader nursing student population, which should be considered when interpreting the findings.

## 4. Discussion

The study aimed to examine the preferences and expectations of nursing students concerning workplace choices in Catalonia, using Herzberg’s motivation‐hygiene theory as the conceptual framework, and analyzes distinctions by gender and generation. The persistent gap between high preferences (> 90%) and low expectations (< 55%) underscores student skepticism about future workplaces amid ongoing shortages threatening care delivery. Hygiene factors (schedules, resources, and contracts) elicited lowest expectations, aligning with international evidence on workload, recognition deficits, and attrition drivers. The findings are especially relevant in the context of the ongoing nursing workforce shortage [4], a condition that continues to challenge healthcare delivery and patient outcomes [18, 19].

The consistently high preferences (> 90% for most items) across all workplace dimensions demonstrate broad consensus among nursing students about essential features of desirable work environments. Even areas with slightly lower agreement, such as reflective/evidence‐based practice, research support via scholarships, and individualized onboarding/progress tracking (80%–88%), still reflect strong valuation of these motivational elements. This uniformity suggests that future nurses share clear ideals for professional fulfillment. In contrast, expectations displayed considerably more variability and remained notably lower across all dimensions (below 55%), highlighting a pronounced difference between motivational factors (dimensions 1 to 3: nursing practice, professional development opportunities, and well‐being and work environment) and hygiene factors (dimensions 4 to 6: Schedules, resources, and terms of employment and remuneration) as students begin their socialization through clinical placements. The consistently lower expectations for hygiene factors, especially scheduling flexibility and work–life balance, where high ratings drop below 20%, suggest students anticipate greater barriers in basic organizational and contractual elements than in more intrinsic, motivational aspects. This pattern reflects findings from international studies, where the work climate, support, stress, and recognition were identified as factors influencing the intention to remain in the profession [20].

The persistent and marked gap between preference and expectation emphasizes a prevailing sense of uncertainty and, in many cases, skepticism or pessimism as students prepare to enter the nursing workforce. This is consistent with previous research providing evidence that poor working conditions, heavy workload [21], and lack of recognition and unsupportive environments [19] are key drivers behind early attrition. Strong preference for flexible work schedules and advance roster notification aligns with evidence that these factors are crucial for retention [22]. Remuneration and professional growth, as underlined in studies from Turkey, China, Spain, and the Czech Republic, further emphasize the global nature of these concerns [23–26], while some national contexts give greater weight to working conditions and schedules [27, 28]. Addressing the preference‐expectation gap requires targeted organizational and policy interventions to better align the realities of nursing practice with the priorities of the emerging workforce.

Gender‐based analysis of nursing students’ workplace preferences and expectations suggests that while overall agreement is strong for motivational factors, women students place greater emphasis on institutional support, personalized care, professional backing, and psychological resources compared to men, aligning with Hoffart et al. [29], who indicated that women value relational, supportive, and flexible workplace structures and express greater concern for issues related to work–life balance and well‐being. For hygienic factors, women consistently rate scheduling flexibility, individualized shifts, and employment stability as more critical than men. Men anticipate higher institutional recognition for advanced nursing practice and permanent employment contracts, corroborating findings by Rajacich et al. [30], who reported that men seek more explicit recognition and clearer advancement pathways. Prosen [31] also observed that male nursing students develop clearer perceptions of future professional roles, which may contribute to persistent gender‐segregated positions. Thus, gender‐aware approaches in nursing education and workforce planning are necessary. Aligning policies and workplace structures with these differentiated needs can narrow the preference‐expectation gap and foster higher retention and satisfaction.

Motivational factors contribute to satisfaction by fulfilling intrinsic needs such as growth and recognition; hygiene factors serve to prevent dissatisfaction by addressing basic workplace conditions like salary and job security. It is imperative to delve deeper into the nature of Herzberg’s framework to comprehend and enhance workplace satisfaction dynamics within the nursing practice context. Some studies have utilized the job demands‐resources model [32] to explore clinical environment’s impact on nurses’ well‐being [33] and to elucidate the role of burnout among nursing staff in the relationship between stress factors and intention to leave the profession, among other outcomes [34].

Generational differences indicate that pre‐Gen Z values support and provide guidance during the transition into the workplace, whereas Gen Z prioritizes autonomy, individualized support, and well‐being. The most pronounced generational differences concern motivational items: Gen Z nurses demand environments that facilitate learning, independence, support, and personal development. Hygienic factors like resources remain important for both generations, showing no significant differences in expectations. Aligning workplace policies with Gen Z expectations is essential for workforce motivation and retention. Research indicates that prioritizing workplace safety, respectful management, role clarity, and fair employment conditions are essential for engaging and retaining Gen Z nurses. Continuous communication and adapting policies to meet their evolving expectations will be critical for future workforce stability and motivation [35]. To attract and retain these young professionals, healthcare organizations must offer personalized benefits that reflect their values, address gaps in decision‐making and communication skills, and create environments where respect, well‐being, and recognition are central [36]. Understanding and responding to these expectations are essential for building a motivated and committed nursing workforce for the future.

The comparison of preference across generations mirrors the patterns identified in gender differences, particularly regarding the significance attributed to well‐being and work environment as motivational aspects, as well as the importance placed on schedules and resources as hygiene factors. It is noteworthy that Gen Z shows lower preferences related to nursing practice and terms of employment and remuneration compared to other generations. However, in terms of expectations, Gen Z ratings are higher across all dimensions. This disparity might suggest a deeper understanding of the healthcare system among the pre‐Gen Z group. This could be attributed to their exposure to traditional healthcare practices and educational approaches. Alternatively, it might reflect a greater awareness of the evolving demands and complexities within the healthcare field, prompting Gen Z to hold higher expectations for improvement in these areas, which might explain their consideration of moving abroad for better opportunities. These findings are consistent with research indicating a perception among Gen Z that the likelihood of securing a good, well‐paying job is low [18].

It is important to highlight that the first three dimensions—nursing practice, professional development opportunities, and well‐being and work environment—align with Herzberg’s motivational factors, as they are intrinsic in nature and contribute to job satisfaction and motivation. The last three dimensions—schedules, resources, and terms of employment and remuneration—correlate with Herzberg’s hygiene factors, as their absence can lead to dissatisfaction, but their presence does not necessarily result in strong motivation. No statistically significant differences were observed between motivational factors and hygiene factors in our study. However, we consider that this conceptual framework, along with the job demands‐resources model, is indispensable for future investigations [32]. Understanding these frameworks is crucial for comprehensively analyzing the work environment and its impact on job satisfaction and performance. Future research should focus on meticulously examining these factors to develop targeted interventions that enhance both intrinsic and extrinsic aspects of the nursing profession, thereby improving overall job satisfaction, reducing burnout, and promoting long‐term retention of nursing staff.

These findings underscore the relevance of Herzberg’s motivational and hygiene factors, alongside the job demands‐resources model, for guiding future research and interventions aimed at enhancing both intrinsic and extrinsic aspects of nursing work, ultimately improving job satisfaction, reducing burnout, and supporting long‐term workforce stability, a critical consideration in the context of ongoing nursing shortages.

## 5. Conclusions

Overall, nursing students’ preferences and expectations show a consistent alignment across motivational and hygiene factors, reinforcing Herzberg’s assertion that both dimensions are essential: motivational factors in fostering satisfaction through intrinsic growth and recognition and hygiene factors in preventing dissatisfaction by ensuring fundamental conditions such as stable schedules and job security [13]. These findings highlight the need for comprehensive workforce strategies that simultaneously address intrinsic and extrinsic aspects of nursing work, rather than relying solely on structural or contractual measures. Further research is needed to establish the value of these frameworks to transcend the traditional approach for analyzing the nursing workforce and foster new perspectives to explore the bottom‐up approach in order to ensure that top‐down strategies will succeed in their implementation [13, 32, 33]. This study represents an initial step in that direction by clarifying how the next generation of nurses prioritizes key motivational and hygiene elements within their future workplaces.

## Author Contributions

All authors contributed to the conceptualization and design of the study. Data collection was carried out by Mireia Subirana‐Casacuberta, Guadalupe Sanchez‐Rueda, Núria Codern‐Bové, Maria Aurelia Sanchez‐Ortega, and Lena Ferrus‐Estopà. All authors contributed to data analysis and interpretation. Mireia Subirana‐Casacuberta and Maria Aurelia Sanchez‐Ortega drafted the initial version of the manuscript.

## Funding

The authors received no financial support for the research, authorship, and/or publication of this article.

## Disclosure

All authors critically reviewed and revised the manuscript for important intellectual content and approved the final version for publication. The corresponding author attests that all listed authors meet authorship criteria and that no others meeting the criteria have been omitted. All authors take responsibility for the integrity of the data and the accuracy of the data analysis.

## Consent

The authors have nothing to report.

## Conflicts of Interest

The authors declare no conflicts of interest.

## Data Availability

The data that support the findings of this study are available on request from the corresponding author. The data are not publicly available due to privacy or ethical restrictions.
